# Influence of sea ice dynamics on population energetics of Western Hudson Bay polar bears

**DOI:** 10.1093/conphys/coaa132

**Published:** 2020-12-30

**Authors:** Amy C Johnson, Jody R Reimer, Nicholas J Lunn, Ian Stirling, David McGeachy, Andrew E Derocher

**Affiliations:** 1 Department of Biological Sciences, University of Alberta, Edmonton, AB T6G 2E9, Canada; 2 Department of Mathematics, University of Utah, Salt Lake City, UT 84112, USA; 3 Environment and Climate Change Canada, CW-422 Biological Sciences Building, University of Alberta, Edmonton, AB T6G 2E9, Canada

**Keywords:** Climate warming, energetics, polar bear, sea ice, *Ursus maritimus*, Western Hudson Bay

## Abstract

The Arctic marine ecosystem has experienced extensive changes in sea ice dynamics, with significant effects on ice-dependent species such as polar bears (*Ursus maritimus*). We used annual estimates of the numbers of bears onshore in the core summering area, age/sex structure and body condition data to estimate population energy density and storage energy in Western Hudson Bay polar bears from 1985 to 2018. We examined intra-population variation in energetic patterns, temporal energetic trends and the relationship between population energetics and sea ice conditions. Energy metrics for most demographic classes declined over time in relation to earlier sea ice breakup, most significantly for solitary adult females and subadult males, suggesting their greater vulnerability to nutritional stress than other age/sex classes. Temporal declines in population energy metrics were related to earlier breakup and longer lagged open-water periods, suggesting multi-year effects of sea ice decline. The length of the open-water period ranged from 102 to 166 days and increased significantly by 9.9 days/decade over the study period. Total population energy density and storage energy were significantly lower when sea ice breakup occurred earlier and the lagged open-water period was longer. At the earliest breakup and a lagged open-water period of 180 days, population energy density was predicted to be 33% lower than our minimum estimated energy density and population storage energy was predicted to be 40% lower than the minimum estimated storage energy. Consequently, over the study, the total population energy density declined by 53% (mean: 3668 ± 386 MJ kg^-1^/decade) and total population storage energy declined by 56% (mean: 435900 ± 46770 MJ/decade). This study provides insights into ecological mechanisms linking population responses to sea ice decline and highlights the significance of maintaining long-term research programs.

## Teaser

We examined intra-population variation in Western Hudson Bay polar bear energetics, temporal trends and relationships with sea ice. We found that energy stored in the population declined from 1985 to 2018 in relation to earlier breakup and longer lagged open-water periods, suggesting multi-year effects of sea ice decline on physiological condition.

## Introduction

Population and ecosystem dynamics are key ecological processes to monitor as ecosystems undergo anthropogenic alterations due to habitat fragmentation and loss (Fahrig, 2003; Mantyka-Pringle *et al.*, 2012) and climate warming (Parmesan and Yohe, 2003; Scheffers *et al.*, 2016). Species have responded to their changing environments through changes in ecological processes including shifts in phenology (Parmesan and Yohe, 2003; Visser and Both, 2005), changes to foraging behaviour (Mahan and Yahner, 1999), altered habitat use/distribution (Mantyka-Pringle *et al.*, 2012; Kortsch *et al.*, 2015) and reduced reproductive and survival rates, with resulting declines in population abundance (Fahrig, 2003; Scheffers *et al.*, 2016). These changes in species’ abundances and distributions can lead to altered community structure and trophic interactions (Rall *et al.*, 2010; Molinos *et al.*, 2015; Scheffers *et al.*, 2016) as well as regime shifts (Petchey *et al.*, 1999; Kortsch *et al.*, 2014), with implications for ecosystem function and stability (de Ruiter *et al.*, 1995; Neutel *et al.*, 2002; Rall *et al.*, 2010). Changes in community structure are especially critical to ecosystems where higher trophic levels are vulnerable to anthropogenic change because altered top predator population dynamics can cause cascading effects (Shackell *et al.*, 2010).

Examining energy dynamics over time can provide insights into ecological responses to both natural and anthropogenic change. Bioenergetics has been studied at individual/species levels using ingestion and assimilation rates (Bailey and Mukerji, 1977; Cressa and Lewis, 1986), prey consumption estimates (Lantry and Stewart, 1993) and metabolism (Lam *et al.*, 1991). Furthermore, broader-scale energetics studies have documented patterns in population energetic requirements (Markussen and Øritsland, 1991; Ryg and Øritsland, 1991; Ernest *et al.*, 2003) and ecosystem energetic dynamics across trophic levels (Sakshaug *et al.*, 1994). Bioenergetics research at various scales is useful for monitoring ecological patterns given that alterations in individual energetic balances may lead to changes in population dynamics (Yodzis and Innes, 1992; Humphries *et al.*, 2004). Thus, understanding temporal dynamics in energetics and relationships to environmental conditions can provide insights into the mechanisms influencing population dynamics and improve our ability to predict how populations respond to future stressors.

The Arctic marine ecosystem has experienced rapid and extensive changes in sea ice in response to climate warming (Comiso, 2002; Stirling and Parkinson, 2006; Stroeve and Notz, 2018; IPCC, 2019). Reduced sea ice extent and earlier sea ice breakup are major factors that affect many Arctic marine species (Comiso, 2002; Stirling and Parkinson, 2006; Meier *et al.*, 2014), especially sea ice-dependent marine mammals, including polar bears (*Ursus maritimus*) (Laidre *et al.*, 2008; Post *et al.*, 2009; Wassmann *et al.*, 2011). Due to their reliance on sea ice for movement, reproduction and as a platform from which to hunt their main prey, ice-associated seals (Stirling and Archibald, 1977; Smith, 1980), polar bears are particularly vulnerable to sea ice decline (Stirling *et al.*, 1999; Stirling and Derocher, 2012). As both a top predator and a species sensitive to sea ice conditions, polar bears are useful for monitoring changing Arctic marine ecosystem dynamics. Long-term research of the Western Hudson Bay (WH) polar bear population, where individuals have been captured, marked and measured over three decades, provides a unique opportunity to examine energetic dynamics relative to sea ice habitat. Declines in WH polar bear body condition (Sciullo *et al.*, 2016), reproductive rates (Stirling *et al.*, 1999), survival (Regehr *et al.*, 2007) and abundance (Lunn *et al.*, 2016) have all been associated with climate warming. Such changes to population dynamics are influenced by individual condition and energy balances (Yodzis and Innes, 1992; Humphries *et al.*, 2004), which in turn are driven by alterations in energy intake and expenditure (Pagano *et al.*, 2018). In Hudson Bay, the open-water period, during which polar bears fast on land, has lengthened (Lunn *et al.*, 2016; Stern and Laidre, 2016) and an increase to a 180-day fasting period is predicted to result in increased starvation and mortality rates (Molnár *et al.*, 2010, 2014; Pilfold *et al.*, 2016). It is therefore important to examine energetic dynamics at various levels (e.g. at the population level as well as within the population) and long-term studies can provide important insights into top predator bioenergetic responses to climate warming and implications for ecosystem dynamics.

Energetics have been examined in polar bear populations using a subjective fat condition index (Stirling *et al.*, 2008), metabolic rates (Pagano *et al.*, 2018), body condition metrics and fasting (e.g. estimated from body mass, body water content and serum biomarkers; Atkinson and Ramsay, 1995; Robbins *et al.*, 2012; Rode *et al.*, 2018) and lipid content (total lipid content quantitatively extracted from adipose tissue; Sciullo *et al.*, 2016). Additionally, the use of body measurements to estimate individual energetic stores can provide insights into energetic dynamics. For example, storage energy and energy density have been used to quantify energy budgets for individual polar bears (Molnár *et al.*, 2009, 2010; Sciullo *et al.*, 2016). Storage energy represents the energy that is available for maintenance, reproduction and growth (i.e. non-structural lipids and proteins), and is influenced by energy intake and expenditure (Molnár *et al.*, 2009, 2010; Sciullo *et al.*, 2016). However, because not all storage energy is available for use when individuals are fasting (due to somatic maintenance), energy density is another useful metric as it accounts for the energy content of storage per unit mass of tissue where energy is needed for maintenance (i.e. the ratio between storage energy and lean body mass; Molnár *et al.*, 2009, 2010; Sciullo *et al.*, 2016). For example, even though adult males have the highest mean storage mass, adult females have higher mean energy density because of their higher fat content of storage and percentage of lipids in adipose tissue (Molnár *et al.*, 2009). These measures are both informative for understanding changes in individual energy balances, as well as predicting changes in population dynamics in response to future conditions.

We used data on annual estimates of the number of bears onshore in the core summering area, age/sex structure and morphometrics collected from WH polar bears to estimate the population energy density and storage energy from 1985 to 2018. Our objectives were to (1) examine temporal dynamics of energy in the WH population, (2) assess the influence of environmental conditions on population energy and (3) explore lagged effects of environmental variables. In addition, we analyzed energy dynamics within the population to provide insights into intra-population variation and examine the vulnerability of different age/sex classes based on energy. This research increases our understanding of the temporal and intra-population energetic patterns of a top marine predator experiencing habitat loss due to climate warming, as well as potential implications for Arctic marine ecosystem dynamics.

## Materials and methods

### Field sampling

**Figure 1:**
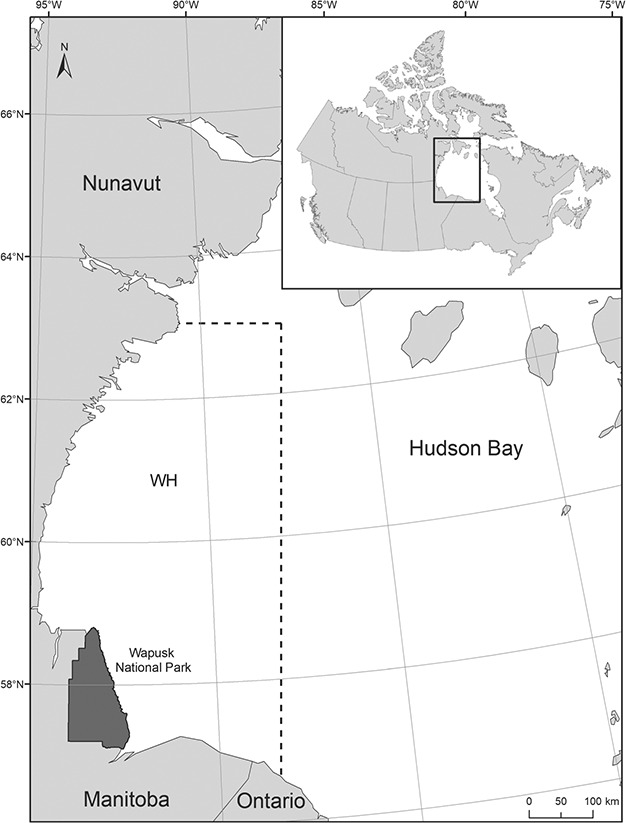
Western Hudson Bay, Canada, where polar bears were captured near the core summering area (Wapusk National Park; indicated in dark grey) from 1985 to 2018. The management boundary of the WH population is indicated by the dashed line.

Hudson Bay is an inland sea that is seasonally ice covered (autumn to spring) and ice-free in summer (Hochheim *et al*., 2010; Fig. 1). When sea ice retreats in summer, WH polar bears come ashore along the western coast of the Bay in northeastern Manitoba, Canada, and remain on land until sea ice freeze-up (Stirling *et al.*, 1999; Lunn *et al.*, 2016). Polar bears were captured in the core summering area of the WH population (Fig. 1) in late August to early October from 1985 to 2018 following standard methods (Stirling *et al.*, 1989). Bears were non-selectively sampled in the order in which they were encountered, independent of age and sex class. Sampling was spread out as evenly as possible between different habitats to account for variation in age/sex class distribution among habitats (e.g. coastal and inland). The timing of autumn captures has remained consistent over the study period (mean day of capture: September 18 for 1985–1989 [SE = 0.37] and September 13 for 2014–2018 [SE = 0.29]). Bears were anesthetized, measured (straight-line body length and axillary girth), marked with uniquely numbered ear tags and tattoos and released. Age was determined from an extracted vestigial premolar (Calvert and Ramsay, 1998) or tooth eruption patterns for dependent offspring. Bears were categorized into seven age, sex and reproductive classes: adult male (≥5 years), solitary adult female (≥5 years), adult female (≥5 years) with offspring, subadult male (2–4 years), subadult female (2–4 years), yearling (ca. 20–22 months) and cub (ca. 8–10 months). All capture and handling techniques were in accordance with the Canadian Council on Animal Care (www.ccac.ca) guidelines and approved by Environment and Climate Change Canada’s Western and Northern Animal Care Committee. Research was conducted under wildlife research permits issued by the Government of Manitoba and the Parks Canada Agency.

### Environmental data

Annual dates of sea ice breakup and freeze-up for the WH management zone were extracted from the mean concentration across 323 grid cells with 25 x 25 km resolution passive microwave satellite raster imagery from the National Snow and Ice Data Center (Cavalieri *et al.*, 1996). The first ordinal date in spring when sea ice concentration was ≤ 50% for three consecutive days was used as the date of sea ice breakup (i.e. the transition from winter to spring, after which bears come ashore), while the first ordinal date in autumn when sea ice was ≥ 10% for three consecutive days was used as the date of freeze-up (i.e. the transition to early winter, when bears move onto the ice; Etkin, 1991; Stirling *et al.*, 1999; Lunn *et al.*, 2016; Castro de la Guardia *et al.*, 2017). The length of the open-water period (i.e. when bears are on land) was calculated as the date of freeze-up minus the date of breakup, then further subtracting 25 days due to the bears arriving onshore approximately 21 to 28 days after breakup (Stirling *et al.*, 1999; Castro de la Guardia *et al.*, 2017; Johnson *et al.*, 2019). In addition, the Arctic Oscillation winter index (AOw) and the North Atlantic Oscillation winter index (NAOw) were extracted for each year to examine broad climate variability. The Arctic Oscillation affects sea ice distribution (Stroeve *et al*., 2011) and is related to polar bear reproduction rates and diet (Derocher, 2005; McKinney *et al.*, 2017), while the North Atlantic Oscillation influences sea ice extent and has been linked to polar bear stress hormones (Bechshøft *et al.*, 2013). AOw was calculated as the mean of January to March Arctic Oscillation index values in each year (National Ocean and Atmospheric Administration; https://www.cpc.ncep.noaa.gov/products/precip/CWlink/daily_ao_index/ao.shtml). NAOw was calculated as the winter index (December to March) from the National Centre for Atmospheric Research (Hurrell, 2012). To account for the influence of environmental conditions of the previous year, we also calculated lagged environmental variables in each year (i.e. the previous year’s sea ice dynamics and climate indices).

### Age/sex class energy patterns

Individual body measurements collected at capture were used to estimate energetic metrics for each bear. Straight-line body length (cm) and axillary girth (cm) were used to estimate body mass (kg) using regression equations in Table 2 from Thiemann *et al*. (2011b) and then energy density (MJ kg^-1^) and storage energy (MJ) were calculated using equations 18 A-E from Molnár *et al*. (2009) (Supplemetary Material Tables S1, S2).

Energy density and storage energy trends over time for each demographic class were analyzed using linear mixed effects models and generalized additive mixed models (GAMMs) with a Gaussian error distribution and identity link function. Analyses were performed on the raw data (i.e. each individual per year) and a random-effect term for individual bear identification number was included to account for repeated sampling. Linear models and GAMMs were compared using Akaike’s Information Criterion (AIC), where ΔAIC < 2 indicated the best model (Supplementary Table S3). Linear models were fit using the *nlme* package (Pinheiro *et al.*, 2020) and GAMMs were fit using the package *gamm4* (Wood and Scheipl, 2020) in R v.4.0.2 (R Core Team, 2020). In addition, linear mixed effects models (Supplementary Table S4; random-effect term for individual bear identification) were defined *a priori* based on ecological hypotheses and were used to assess the relationship between energy density or storage energy for each class and the environmental variables (sea ice breakup, length of the open-water period, AOw, NAOw and lagged effects). Environmental variables were assessed for collinearity and variables that were correlated (*r* > |0.6|) were not included in the same model (Supplementary Table S5). Model selection was conducted using AIC.

As the energy density and storage energy values were non-normally distributed (Shapiro–Wilk test, *P* ≤ 0.05) and standard transformations did not improve normality, we used Kruskal–Wallis ANOVA and Dunn’s non-parametric tests to examine differences among age/sex classes.

### Estimating population energy density and storage energy

Total population energy density and storage energy were calculated based on population structure, annual estimates of the number of bears onshore in the core summering area and individual body measurements. Capture records from 1985 to 2018 were used to estimate population structure; however, variation in yearly sample sizes (e.g. low numbers of bears caught from certain age classes in certain years) necessitated the use of bootstrapping over a five-year moving window to estimate yearly percentages of each age/sex class. Bootstrapping was used to incorporate uncertainty into parameter estimates by resampling with replacement from the values in the capture record and then calculating the mean parameter value from the resampled values (Fortune *et al.*, 2013; Laidre *et al.*, 2020). Therefore, step one of the population energy estimation process (Supplementary Table S6) involved calculating the mean percentage of each class in the five-year window around the year of interest from 2000 bootstrap iterations (sampling with replacement from the percentage of bears in each class in each year from the five-year period) using the *boot* package in R (Canty and Ripley, 2019) to represent yearly population structure.

A previous abundance estimate for WH (Lunn *et al.*, 2016) used Bayesian multistate capture-recapture models with dead-recovery and live-recapture data; however, dead-recovery data were not available post-2011. Therefore, annual estimates of the number of bears onshore in the core summering area for this study were calculated in the program MARK (Cooch and White, 2015) using the POPAN formulation (Schwarz and Arnason, 1996; Supplementary Material). There was a significant correlation between these estimates and those from Lunn *et al*. (2016) (Pearson correlation, coefficient = 0.79, *P* < 0.001), and we consider the Bayesian and MARK analyses equally valid approaches. To account for uncertainty in MARK estimates, step two involved drawing a random value from a normal distribution (based on the MARK values) to estimate the annual number of bears onshore. The numbers of bears of each class were then calculated in step three by multiplying the bootstrapped age/sex class structure by the estimated annual number of bears onshore.

In step four, the yearly mean energy density and storage energy of an individual bear in each class were calculated from 2000 bootstrap iterations (sampling with replacement from the energy values of bears in that class in the year of interest) using the *boot* package in R (Canty and Ripley, 2019). Step five involved calculating the yearly total energy density and storage energy for each class by multiplying the number of bears in that class by the mean energy of that class.

In step six, the yearly total population energy density and storage energy were calculated by summing the energy values across classes. To account for uncertainty in this process, steps steps one to six were conducted 10,000 times and the resulting mean and standard error of the mean (SE) were used as the total population energy density and storage energy estimates in further analyses.

### Temporal dynamics of population energy and environmental analyses

We examined temporal trends (1985–2018) in total population energy density, storage energy and temporal dynamics of sea ice variables by comparing linear regression models and generalized additive models using AIC. We used multiple linear regression analysis to examine the relationship between total population energy values and environmental variables (Supplementary Table S4). Model selection was conducted using AIC and the best model was used to make predictions about population energy given potential future environmental conditions (i.e. 180 day fasting period; Molnár *et al*., 2010, 2014; Pilfold *et al*., 2016). Statistical analyses were conducted in R v.4.0.2 (R Core Team, 2020).

## Results

There were 4346 captures of 2533 individual bears from 1985 to 2018, with a mean of 128 bears (SE = 11) captured/year (Supplementary Table S7). There were 1159 adult male, 540 solitary adult female, 807 adult female with offspring, 296 subadult male, 331 subadult female, 393 yearling and 820 cub captures (Supplementary Table S8). The mean number of captures of the same individual was 1.7 (range = 1–12).

Sea ice breakup varied from 17 May (2015) to 10 July (1992) and occurred significantly earlier from 1985 to 2018, with mean breakup occurring 5.5 days/decade earlier (linear regression, *P* ≤ 0.05; Supplementary Fig. S1). Sea ice freeze-up varied from 4 November (1993) to 7 December (2016) and occurred significantly later over time, with mean freeze-up occurring 4.3 days/decade later (linear regression, *P* ≤ 0.001; Supplementary Fig. S1). The length of the open-water period varied from 102 days (1992) to 166 days (2015) and significantly lengthened over time, with a mean increase of 9.9 days/decade (linear regression, *P* ≤ 0.001; Supplementary Fig. S1).

### Age/sex class energy patterns

**Figure 2:**
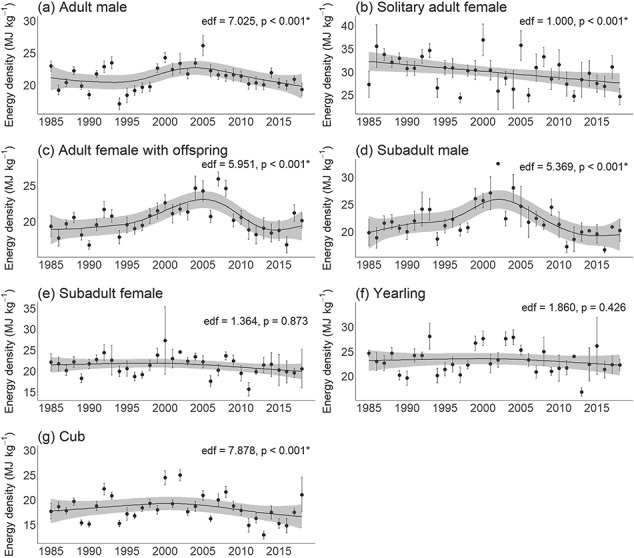
Generalized additive models (black line) with 95% confidence intervals (grey) for energy density (mean ± standard error) over time for each age/sex class of WH polar bears. See Table S9 for model summaries. edf: effective degrees of freedom (where an edf of 1.0 indicates a linear trend and an edf > 1.0 indicates a non-linear trend).

**Figure 3:**
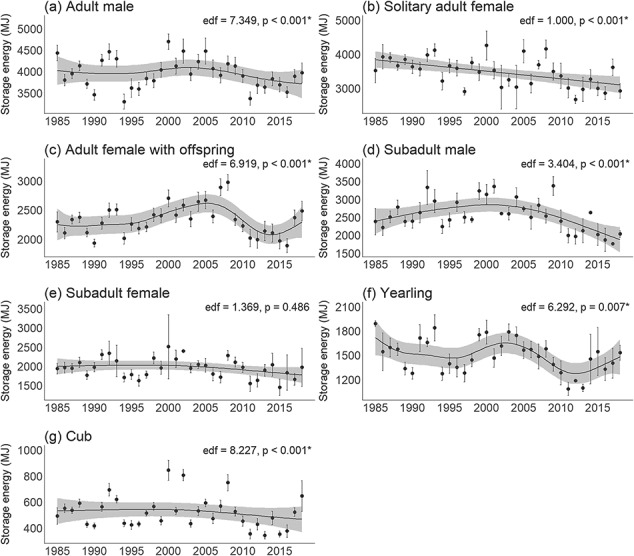
Generalized additive models (black line) with 95% confidence intervals (grey) for storage energy (mean ± standard error) over time for each age/sex class of WH polar bears. See Table S10 for model summaries. edf: effective degrees of freedom (where an edf of 1.0 indicates a linear trend and an edf > 1.0 indicates a non-linear trend).

Energy density significantly declined linearly over time for solitary adult females (mean: 1.7 MJ kg^-1^/decade) (generalized additive mixed model, effective degrees of freedom [edf] = 1.000, *P* < 0.001; linear mixed effects model, *P* < 0.001), significantly varied non-linearly over time for adult males, adult females with offspring, subadult males and cubs (generalized additive mixed model, edf > 1.000, *P* < 0.05), and did not significantly vary over time for subadult females and yearlings (generalized additive mixed model, edf > 1.000, *P* > 0.05; Fig. 2; Supplementary Table S9). Storage energy significantly declined linearly over time for solitary adult females (mean: 232 MJ/decade) (generalized additive mixed model, edf = 1.000, *P* < 0.001; linear mixed effects model, *P* < 0.001), significantly varied non-linearly over time for adult males, adult females with offspring, subadult males, yearlings, and cubs (generalized additive mixed model, edf > 1.000, *P* < 0.05), and did not significantly vary over time for subadult females (generalized additive mixed model, edf > 1.000, *P* > 0.05; Fig. 3; Supplementary Table S10). Furthermore, there was a significant decline in the contribution of subadult males (mean: 1.3%/decade) to total population storage energy over time (linear regression, *P* = 0.015), while adult males significantly increased (mean: 3.2%/decade) in their contribution to total population storage energy over time (linear regression, *P* = 0.021) (Supplementary Fig. S2; Table S11).

**Figure 4:**
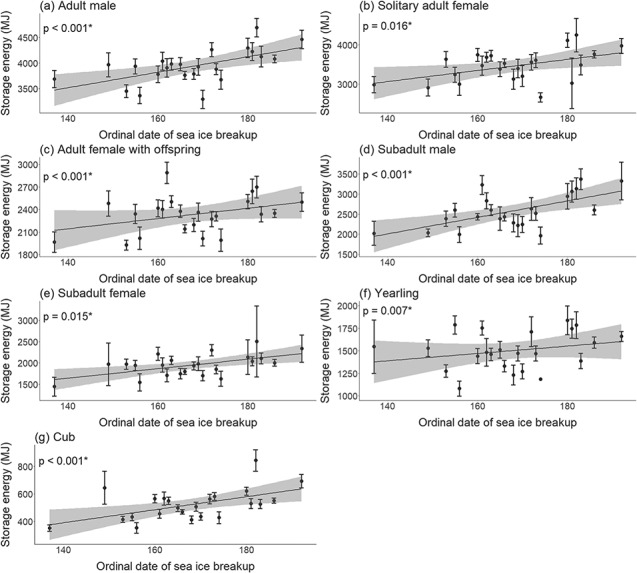
Linear regressions (black line) with 95% confidence intervals (grey) for storage energy (mean ± standard error) with the date of sea ice breakup for each age/sex class of WH polar bears. See Table S15 for model summaries.

**Figure 5:**
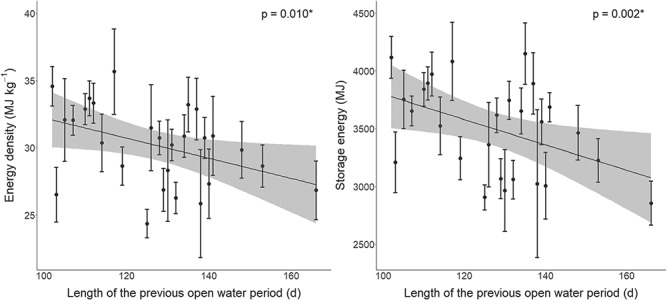
Linear regressions (black line) with 95% confidence intervals (grey) for solitary adult female energy density (left; mean ± standard error) and storage energy (right) with the length of the previous open-water period. See Tables S13 and S15 for model summaries.

Energy density and storage energy for all classes were significantly lower when sea ice breakup dates were earlier (linear mixed effects model, *P* < 0.05; Fig. 4 and Supplementary Fig. S3; Tables S12–S15). A longer lagged open-water period was associated with significantly reduced energy density and storage energy for solitary adult females (linear mixed effects model, *P* = 0.010, 0.002, respectively; Fig. 5), while there were no significant declines for the other classes (linear mixed effects model, *P* > 0.05; Supplementary Figs S4, S5; Tables S13, S15).

Energy density was significantly different among classes (Kruskal–Wallis, χ^2^ = 958.3, df = 6, *P* < 0.001). Solitary adult females had significantly higher energy density than all other classes (Dunn’s test, *P* ≤ 0.05; Supplementary Tables S8, S16). Cubs and adult females with offspring had significantly lower energy density than all other classes, while adult males, subadult males/females and yearlings had intermediate energy density. Storage energy was also significantly different among classes (Kruskal–Wallis, χ^2^ = 3398.2, df = 6, *P* < 0.001). Adult males had significantly higher storage energy than all other classes, followed by solitary adult females (Dunn’s test, *P* ≤ 0.05; Supplementary Tables S8, S17). Subadult males/females and adult females with offspring had intermediate storage energy. Cubs and yearlings had significantly lower storage energy than all other classes.

### Temporal dynamics of population energy

**Figure 6:**
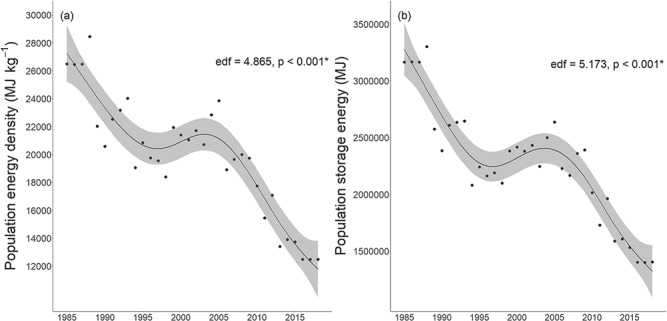
Generalized additive models (black line) with 95% confidence intervals (grey) for estimated total population energy density (a) and population storage energy (b) for WH polar bears from 1985 to 2018. See Table S18 for model summaries. edf: effective degrees of freedom (where an edf of 1.0 indicates a linear trend and an edf > 1.0 indicates a non-linear trend).

From 1985 to 2018, the total population energy density significantly varied non-linearly (generalized additive model, edf = 4.9, *P* < 0.001; Fig. 6; Supplementary Table S18) and declined by 53% (mean: 3668 ± 386 MJ kg^-1^/decade). Similarly, total population storage energy significantly varied non-linearly over time (generalized additive model, edf = 5.2, *P* < 0.001; Fig. 6; Supplementary Table S18) and declined by 56% (mean: 435900 ± 46770 MJ/decade).

### Population energy and the environment

**Figure 7:**
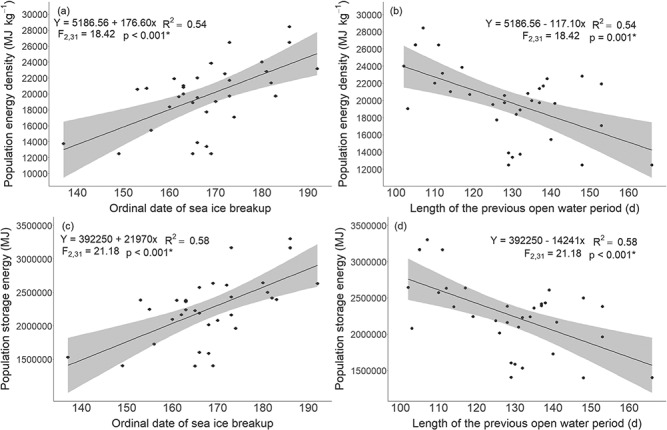
Linear regressions (black line) with 95% confidence intervals (grey) for estimated total population energy density (a, b) and storage energy (c, d) with sea ice breakup and the length of the previous open-water period (lagged by one year) for WH polar bears from 1985 to 2018.

The best models for population energy density and storage energy included sea ice breakup and the lagged open-water period, while AOw, NAOw and their lagged effects were not included in the best models (Supplementary Table S19). Total population energy density was significantly lower when sea ice breakup occurred earlier and the lagged open-water period was longer (multiple linear regression, *P* < 0.001, *P* = 0.001, respectively; Fig. 7, Table 1). The best multiple linear regression model predicted that at the earliest observed breakup (ordinal date 137) and 180 day lagged open-water period, total population energy density would be 8303 MJ kg^-1^ (33% lower than the minimum energy density value that was calculated in our study, 12475 MJ kg^-1^).

**Table 1:** The best multiple regression models for total population energy density and storage energy with the environmental covariates for WH polar bears from 1985 to 2018. The model F-statistic, R^2^, β coefficients (β), standard error (SE) and *P*-values (*P*) are included. Model number corresponds to Supplementary Table S4. ^*^Significant, *P* ≤ 0.05.

Response	Model no.	Covariates	F	R^2^	Intercept β	β	SE	*P*
Energy density	7	Breakup	18.42	0.54	5186.56	176.60	45.82	<0.001*
		OpenWater_Lag				-117.10	33.59	0.001*
Storage energy	7	Breakup	21.18	0.58	392250	21970	5261	<0.001*
		OpenWater_Lag				-14241	3857	<0.001*

Similarly, total population storage energy was significantly lower when sea ice breakup occurred earlier and the lagged open-water period was longer (multiple linear regression, *P* < 0.001, 0.001, respectively; Fig. 7, Table 1). At the earliest breakup (ordinal date 137) and 180 day lagged open-water period, population storage energy was predicted to be 838781 MJ (40% lower than our minimum estimated storage energy, 1398529 MJ).

## Discussion

We examined intra-population variation in energy density and storage energy, temporal dynamics in energetics and the influence of sea ice dynamics on WH polar bear population energetics from 1985 to 2018. We found temporal variation in energetic dynamics among age/sex classes. Solitary adult females showed decreases in energy density and storage energy over time while subadult males declined in their contribution to total population storage energy over time, indicating the greater vulnerability of these classes to future environmental changes. Decreases in storage energy indicate that bears had less energy available for maintenance, growth and survival (Molnár *et al.*, 2009; Sciullo *et al.*, 2016). Adult females and juveniles are often vulnerable demographic groups and their condition can influence population trends by affecting reproduction and survival rates (Lockyer, 1986; Miller *et al.*, 2011; Fortune *et al.*, 2013; Keay *et al.*, 2018). The small body size, dietary constraints, energetic demands of growth, risk of kleptoparasitism from larger bears and inexperienced hunting skills of younger bears make them more vulnerable to reductions in sea ice and thus prey availability (Stirling, 1974; Rode *et al.*, 2010; Thiemann *et al.*, 2011a; Pilfold *et al.*, 2016; Johnson *et al.*, 2019; Laidre *et al.*, 2020). In contrast, adult males can best buffer against sub-optimal conditions given their larger body size, broader diets, more effective hunting skills and kleptoparasitism from smaller bears (Stirling, 1974; Regehr *et al.*, 2007; Thiemann *et al.*, 2011a; Pilfold *et al.*, 2016; Johnson *et al.*, 2019). These patterns highlight the importance of continued monitoring of the condition of young bears.

The reproductive status of adult female polar bears in WH influenced their energy patterns. Solitary adult females had higher energy density and storage energy than adult females with offspring, but solitary females experienced significant declines in both energy metrics over time whereas females with offspring had lower but relatively stable energy values. These results are consistent with observations that solitary adult females have higher body condition due to their accumulation of body fat in preparation for the energetic requirements of gestation and lactation (Atkinson and Ramsay, 1995; Thiemann *et al.*, 2006; Sciullo *et al.*, 2016). The maternity denning period in WH involves up to eight months of fasting (Ramsay and Stirling, 1988) and the amount of energy a solitary adult female accumulates before denning determines the likelihood of successfully producing cubs, as well as subsequent cub survival (Derocher and Stirling, 1994, 1996, 1998; Atkinson and Ramsay, 1995) and litter size (Laidre *et al.*, 2020). Decreases in solitary adult female condition can therefore translate into a decline in cub production, cub survival and reproductive success, which have already been documented in WH (Derocher and Stirling, 1995; Stirling *et al.*, 1999). The observed declines in solitary adult female energy may reflect increased difficulty over time in accumulating sufficient resources. In contrast, females with offspring have lower energy reserves due to ongoing lactational energetic demands that make the accumulation and storage of energy more difficult (Derocher *et al.*, 1993; Arnould and Ramsay, 1994; Atkinson and Ramsay, 1995). There is likely a threshold of energetic reserves that is required to successfully reproduce (Molnár *et al.*, 2010; Reimer *et al.*, 2019). For instance, Derocher *et al*. (1992) found that the lowest weight of an adult female known to have successfully reproduced was 189 kg, Robbins *et al*. (2012) indicated that females require 20% body fat when entering a den to be able to successfully produce cubs, and Reimer *et al*. (2019) suggested a reproductive threshold for energy density of ~14 MJ kg^-1^. Similarly, our results indicated that adult females with offspring had relatively stable energy density (minimum: 7.9 MJ kg^-1^, median: 19.8 MJ kg^-1^; Fig. 2) and storage energy (minimum: 916 MJ; median: 2241 MJ; Fig. 3), suggesting energetic thresholds for reproduction. Similar to Robbins *et al*. (2012), our results suggest the vulnerability of females with offspring to nutritional stress due to their low energetic reserves, as well as the sensitivity of solitary adult females that need sufficient energy to reproduce.

Our study also demonstrated the association between age/sex class energetic patterns and environmental conditions. Reduced energy density and storage energy were associated with earlier sea ice breakup and this relationship was significant for all classes. These results are consistent with the relationship between earlier breakup and reduced body condition in WH (Stirling *et al.*, 1999; Sciullo *et al.*, 2016). Our finding that the lagged open-water period was an important predictor for solitary adult female energy density and storage energy suggests that the previous year’s sea ice conditions may influence the ability of solitary females to accumulate energy reserves in preparation for reproduction. Similarly, Derocher and Stirling (1994) found that an adult female’s condition in the previous year was a strong determining factor for reproductive success in WH. In other polar bear populations, lower body condition has been associated with time lags in breakup date and the duration of the ice-free period (Galicia *et al.*, 2019; Laidre *et al.*, 2020). The observed decline in solitary adult female energy and the relationship with the lagged open-water period suggests that females may not be able to recover from declines in stored energy that have occurred in previous years, which has the potential to accumulate over time and affect lifetime reproductive success. As cub survival has declined in relation to earlier breakup (Regehr *et al.*, 2007), a factor potentially contributing to the decline in energy metrics for solitary adult females is the addition to this class of non-pregnant females in poor condition that lost cubs. A limitation of our study is an inability to distinguish between pregnant and non-pregnant solitary females, as well as differences in the probability of detecting each during the on-land period. Overall, our results indicate that polar bear energetic balances are negatively affected by sea ice declines and that vulnerable demographic groups include younger bears and adult females.

The WH population declined from approximately 1185 to 806 bears from 1987 to 2011 (Lunn *et al.*, 2016); furthermore, WH body condition has also declined over time, including storage energy declines from 2004 to 2013 (Derocher and Stirling, 1995; Stirling *et al.*, 1999; Sciullo *et al.*, 2016). Our results provide new insights into long-term population-level trends as we found that WH total population energy density and storage energy declined significantly over the 34 year study. Reduced population abundance in addition to declining body condition of individuals both contribute to the observed decline in the total energy stored in this population. However, it is difficult to isolate the relative contributions of individual changes in body condition with population-level energy dynamics because of the relationship between these factors. Nonetheless, declines in individual energy balances and subsequent consequences for survival and reproduction illustrate the mechanism linking climate change and population dynamics (Yodzis and Innes, 1992; Humphries *et al.*, 2004; Molnár *et al.*, 2009, 2010; Pagano *et al.*, 2018). Understanding the ecological mechanisms behind demographic change is important for wildlife management and can improve our predictions about how populations may respond to future climate warming (Cherry *et al*., 2009; Pagano *et al*., 2018; Reimer *et al*., 2019).

We found that WH experienced significant long-term change in sea ice dynamics, with a lengthening of the open-water period by approximately 9.9 days/decade. WH polar bear population energy density and storage energy were both significantly reduced when sea ice breakup was earlier and the lagged open-water period was longer, demonstrating a linkage between declining sea ice and reduced energetic balances. Sea ice is the most important single factor influencing polar bear demographic responses in the changing Arctic marine ecosystem (Stirling *et al.*, 1999; Bromaghin *et al.*, 2015; Lunn *et al.*, 2016). Our results are consistent with the association between earlier breakup/later freeze-up and declining body condition (Stirling *et al.*, 1999; Obbard *et al.*, 2016; Sciullo *et al.*, 2016; Laidre *et al.*, 2020), increased stress (Boonstra *et al.*, 2020), altered foraging ecology (McKinney *et al.*, 2009; Johnson *et al.*, 2019), and reduced reproduction/survival rates and abundance (Regehr *et al.*, 2007; Rode *et al.*, 2010; Lunn *et al.*, 2016; Obbard *et al.*, 2018) reported in various polar bear populations including WH, Southern Beaufort Sea, Southern Hudson Bay and Baffin Bay. Changes to energetic intake and expenditure in response to sea ice dynamics have consequences for energetic balances (Pagano *et al.*, 2018). Polar bear energetic intake is reduced when breakup occurs earlier and freeze-up occurs later because the spring hunting period is shortened and bears are forced to fast on land for longer periods in poorer condition (Cherry *et al*., 2009, 2013; Rode *et al*., 2014, 2018). Meanwhile, energetic expenditure increases due to declines in optimal habitat (Durner *et al.*, 2009; Stern and Laidre, 2016), increasingly fragmentated and drifting sea ice (Mauritzen *et al.*, 2003; Sahanatien and Derocher, 2012; Auger-Méthé *et al.*, 2016; Durner *et al.*, 2017), and long-distance swims as a result of more open water (Durner *et al.*, 2011; Pagano *et al.*, 2012; Pilfold *et al.*, 2017). We found that the open-water period increased from 105 days in 1985 to 145 days in 2018, with a maximum of 166 days in 2015. An increase in the fasting period from 120 days to 165 days was predicted to lead to higher starvation rates for adult male polar bears in WH (Robbins *et al.*, 2012), while fasts >180 days were predicted to lead to additional increases in starvation-related mortality (Molnár *et al.*, 2010, 2014; Pilfold *et al.*, 2016). Similarly, our predictions indicated that at 180 day previous fasting period, population energy density and storage energy would be 33% and 40% lower than the minimum estimated values, respectively. Decreases in the length of the spring foraging period are predicted to lead to declines in female polar bear expected fitness (Reimer *et al.*, 2019) and higher fasting rates have occurred concurrently with reductions in survival and abundance (Cherry *et al.*, 2009; Rode *et al.*, 2014, 2018). Our predicted declines in WH population energy at longer fasting periods have implications for population vital rates. Moreover, the importance of the lagged open-water period suggests that there are cumulative effects of prior conditions that affect the ability of bears to recover from nutritional stress, similar to the lag effect observed for a WH polar bear stress biomarker (Boonstra *et al.*, 2020). In response to predicted future sea ice decline, WH polar bears are at risk of further declines to energetic balances leading to reduced survival rates for young bears and decreased reproductive success, which may ultimately result in a functionally extinct population (Castro de la Guardia *et al.*, 2013; Pilfold *et al.*, 2016).

The Arctic marine ecosystem has already experienced various alterations due to climate warming-induced sea ice decline, such as changes to community structure and regime shifts, which can influence ecosystem structure and function (Wassmann *et al.*, 2011; Kortsch *et al.*, 2014; Post *et al.*, 2019; Huntington *et al.*, 2020). Habitat loss is a primary factor influencing biodiversity (Brook *et al.*, 2008; Mantyka-Pringle *et al.*, 2012) and in the Arctic marine ecosystem, loss of sea ice habitat has been associated with changes in Arctic marine mammal populations including altered movements, foraging and life history events (Laidre *et al.*, 2008; Post *et al.*, 2019). In addition, our observed decline in population energy of a top predator has implications for ecosystem dynamics. Changing ecological dynamics can be driven by lower trophic level trends (Brown *et al.*, 2018; Waga *et al.*, 2019) as well as top predator dynamics (Pace *et al.*, 1999; Schmitz *et al.*, 2000; Frank *et al.*, 2005; Huntington *et al.*, 2020). Altered top predator population dynamics may cascade through ecosystems and influence trophic interactions and food web dynamics (Pace *et al.*, 1999; Schmitz *et al.*, 2000; Frank *et al.*, 2005). For example, reduced body size of top predators has been associated with a weakening of predation pressure on lower trophic levels (Shackell *et al.*, 2010). A potential consequence of reduced WH polar bear energetic balances is therefore altered trophic interactions with their primary prey species, ringed seals (*Pusa hispida*). However, Hudson Bay ringed seals have similarly shown population declines over time (Young *et al.*, 2015; Ferguson *et al.*, 2017) and ringed seal body condition trends can be related to a complex interaction between climate indices and local sea ice conditions (Harwood *et al.*, 2020); thus, our limited understanding of changing predator-prey interactions and energy dynamics in the Arctic would benefit from long-term monitoring of ecological parameters across multiple trophic levels (Yurkowski *et al.*, 2020). As future sea ice declines threaten Arctic wildlife populations (Post *et al.*, 2019; Hwang *et al.*, 2020), examining trends in various aspects of apex predator ecology at multiple scales can be a useful monitoring approach. As the Arctic continues to warm, polar bears can act as an indicator species to improve our understanding of changing ecosystem dynamics (Rode *et al.*, 2018). Our research reinforces the importance of long-term monitoring of individual physiological condition and broad population patterns.

## Author contributions

All authors contributed to the study design and collected the data, ACJ and JRR contributed to model design, ACJ analyzed the data and wrote the first draft of the manuscript, and all authors contributed to revisions.

## Funding

This work was supported by the Alberta Society of Professional Biologists [A.C.J.]; Canadian Association of Zoos and Aquariums; Canadian Circumpolar Institute; Canadian Wildlife Federation; Care for the Wild International; Churchill Northern Studies Centre; Earth Rangers Foundation; Environment and Climate Change Canada; Hauser Bears; the Isdell Family Foundation; Kansas City Zoo; Manitoba Water Stewardship and Biodiversity Division; Natural Sciences and Engineering Research Council of Canada [A.C.J., A.E.D., I.S.]; Northern Science Training Program [A.C.J.]; Parks Canada Agency; Pittsburgh Zoo Conservation Fund; Polar Bears International; Quark Expeditions; Schad Foundation; the Takla Foundation; University of Alberta; W. Garfield Weston Foundation; Wildlife Media Inc.; and World Wildlife Fund Canada.

## Supplementary Material

Supplementary_Material_coaa132
