# Supplementary material for: Influence of sea ice dynamics on population energetics of Western Hudson Bay polar bears
Source: Conserv Physiol. 2020 Dec 30;8(1):coaa132. doi: 10.1093/conphys/coaa132 (PMC7772618; doi:10.1093/conphys/coaa132)
Supplement: Supplementary_Material_coaa132 [file supplementary_material_coaa132.docx]

**Influence of sea ice dynamics on population energetics of Western Hudson Bay polar bears**

Amy C. Johnson*^1^, Jody R. Reimer^1,2^, Nicholas J. Lunn^3^, Ian Stirling^1,3^, David McGeachy^3^, Andrew E. Derocher^1^

^1^ Department of Biological Sciences, University of Alberta, Edmonton, AB, Canada T6G 2E9

^2^ Department of Mathematics, University of Utah, Salt Lake City, UT, USA 84112

^3^ Environment and Climate Change Canada, CW-422 Biological Sciences Building, University of Alberta, Edmonton, AB, Canada T6G 2E9

* ***Corresponding author***: Amy C. Johnson. acj1@ualberta.ca

**Supplementary Methods**

**Estimating population energy density and storage energy**

*The program MARK*

We used the program MARK (Cooch and White, 2015) to estimate the annual numbers of polar bears onshore in the core summering area using the POPAN formulation (Schwarz and Arnason, 1996) and data from the capture and release of polar bears collected by Environment and Climate Change Canada (e.g., Ramsay and Stirling, 1988; Derocher and Stirling, 1995; Stirling *et al.*, 1999; Regehr *et al.*, 2007; Lunn *et al.*, 2016) and from conflict bears captured by Government of Manitoba near the community of Churchill (Kearney, 1989). This method uses apparent survival, probability of detection, and the Probability of Entrance (PENTS) to estimate the super-population (N) for which subsequent abundance estimates N*_I_* are derived parameters where the standard error is calculated using the Delta method. Captures were grouped by sex, bears greater or equal to one year of age were used, and estimates of cubs were included following methods by Lunn *et al*. (1997). *A priori* models consisted of a full time dependent model, both ɸ and p time-invariant, a ɸ time-invariant and p time-invariant model, and a ɸ time-invariant for males/time-variant for females with p time-variant. Model fit was assessed using median ĉ estimated from the chi square of Test 2 + Test 3 from program release divided by the degrees of freedom. The estimate of ĉ was > than 1 and to account for lack of fit, model adjustments were made and ĉ was adjusted to 1.68. We used QAICc to select the top model. The top model carried 99.8% of the model weight and included constant survival and time varying probability of detection.

**References**

Cooch EG, White GW (2015) Program MARK: A Gentle Introduction. 14th ed. Colorado State University, Fort Collins, CO, USA.

Derocher AE, Stirling I (1995) Temporal variation in reproduction and body mass of polar bears in western Hudson Bay. *Can J Zool* 73: 1657–1665.

Kearney S (1989) The polar bear alert program in Churchill, Manitoba. In: Bromley, M. (Ed.), Bear-People Conflicts: Proceedings of a Symposium on Management Strategies. Northwest Territories Department of Renewable Resources, Yellowknife, Northwest Territories, Canada. pp 83–92.

Lunn NJ, Servanty S, Regehr EV, Converse SJ, Richardson E, Stirling I (2016) Demography of an apex predator at the edge of its range: impacts of changing sea ice on polar bears in Hudson Bay. *Ecol Appl* 26: 1302–1320.

Lunn NJ, Stirling I, Andriashek D, Kolenosky GB (1997) Re-estimating the size of polar bears population in western Hudson Bay. *Arctic* 50: 234–240.

Molnár PK, Klanjscek T, Derocher AE, Obbard ME, Lewis MA (2009) A body composition model to estimate mammalian energy stores and metabolic rates from body mass and body length, with application to polar bears. *J Exp Biol* 212: 2313–2323.

Ramsay MA, Stirling I (1988) Reproductive biology and ecology of female polar bears (*Ursus maritimus*). *J Zool* 214: 601–633.

Regehr EV, Lunn NJ, Amstrup SC, Stirling I (2007) Effects of earlier sea ice breakup on survival and population size of polar bears in Western Hudson Bay. *J Wildl Manage* 71: 2673–2683.

Schwarz CJ, Arnason AN (1996) A general methodology for the analysis of capture-recapture experiments in open populations. *Biometrics* 52: 860–873.

Stirling I, Lunn NJ, Iacozza J (1999) Long-term trends in the population ecology of polar bears in western Hudson Bay in relation to climatic change. *Arctic* 52: 294–306.

Thiemann GW, Lunn NJ, Richardson ES, Andriashek DS (2011) Temporal change in the morphometry-body mass relationship of polar bears. *J Wildl Manage* 75: 580–587.

**Supplementary Tables**

Table S1. Descriptions of parameters used to estimate Western Hudson Bay polar bear energy density and storage energy.

| Parameter | Description and units |
| --- | --- |
| AXG | Axillary girth (cm) |
| SLEN | Straight-line body length (cm) |
| M | Body mass (kg) |
| L | Straight-line body length (m) |
| E | Storage energy (MJ) |
| M_STO_ | Storage mass (kg) |

Table S2. Equations used to estimate polar bear energy density and storage energy.

| Parameter and units | Equation | Source |
| --- | --- | --- |
| Body mass (kg) from 1985 – 1996 | 0.00011205 x AXG^1.977^ x SLEN^0.931^ | (Thiemann et al., 2011) |
| Body mass (kg) from 1997 – 2018 | 0.00010309 x AXG^1.729^ x SLEN^1.179^ | (Thiemann et al., 2011) |
| Cub storage energy (MJ) | 20.77M – 310.30L^3^ | (Molnár et al., 2009) |
| Yearling storage energy (MJ) | 25.84M – 386.05L^3^ | (Molnár et al., 2009) |
| Subadult storage energy (MJ) | 24.97M – 373.05L^3^ | (Molnár et al., 2009) |
| Adult female storage energy (MJ) | 26.14M – 390.53L^3^ | (Molnár et al., 2009) |
| Adult male storage energy (MJ) | 19.50M – 291.33L^3^ | (Molnár et al., 2009) |
| Storage mass (kg) | M – 14.94L^3^ | (Molnár et al., 2009) |
| Energy density (MJ kg^-1^) | E / (M – M_STO_) | (Molnár et al., 2009) |

Table S3. Comparisons of linear models and generalized additive models. Model selection was conducted using Akaike’s Information Criterion (AIC) and the best model is indicated in bold. If the linear model was the best model or if the linear model and generalized additive model were within 4 AIC (indicated by an asterisk), then both the linear model and generalized additive model summaries were provided in Tables S9, S10, and S11.

| Model | Linear model AIC | Generalized additive model AIC | ΔAIC |
| --- | --- | --- | --- |
| Adult male energy density over time | 6945.696 | **6909.678** | 36.018 |
| Solitary adult female energy density over time | 3778.533 | **3775.955** | 2.578* |
| Adult female with offspring energy density over time | 4944.178 | **4908.653** | 35.525 |
| Subadult male energy density over time | 1858.662 | **1839.094** | 19.568 |
| Subadult female energy density over time | 2036.431 | **2033.991** | 2.44* |
| Yearling energy density over time | **2506.754** | 2509.401 | 2.647 |
| Cub energy density over time | 4997.328 | **4967.688** | 29.64 |
|  |  |  |  |
| Adult male storage energy over time | 19224.15 | **19205.51** | 18.64 |
| Solitary adult female storage energy over time | 8906.637 | **8904.059** | 2.578* |
| Adult female with offspring storage energy over time | 12574.06 | **12540.69** | 33.37 |
| Subadult male storage energy over time | 4762.188 | **4743.681** | 18.507 |
| Subadult female storage energy over time | 5101.236 | **5098.769** | 2.467* |
| Yearling storage energy over time | 5911.874 | **5894.698** | 17.176 |
| Cub storage energy over time | 11051.390 | **11007.460** | 43.93 |
|  |  |  |  |
| Adult male storage energy contribution over time | 237.359 | **233.471** | 3.888* |
| Solitary adult female storage energy contribution over time | 230.519 | **219.496** | 11.023 |
| Adult female with offspring storage energy contribution over time | 191.038 | **185.667** | 5.371 |
| Subadult male storage energy contribution over time | **173.601** | 173.829 | 0.228 |
| Subadult female storage energy contribution over time | **174.436** | 175.520 | 1.084 |
| Yearling storage energy contribution over time | **164.322** | 165.979 | 1.657 |
| Cub storage energy contribution over time | **111.961** | 112.897 | 0.936 |
|  |  |  |  |
| Population energy density over time | 624.128 | **585.755** | 38.373 |
| Population storage energy over time | 950.227 | **888.363** | 61.864 |

Table S4. List of *a priori* multiple linear regression models. The response variable was either energy density or storage energy of polar bears in the Western Hudson Bay population. Explanatory covariates included combinations of large scale atmospheric indices and local sea ice conditions (and lagged effects): Breakup (date of sea ice breakup), NAOw (winter North Atlantic Oscillation), AOw (winter Arctic Oscillation), OpenWater_Lag (length of the previous open water period), NAOw_Lag (previous winter North Atlantic Oscillation), and AOw_Lag (previous winter Arctic Oscillation).

| Model no. | No. covariates | Model structure |
| --- | --- | --- |
| 1 | 4 | Breakup + NAOw + OpenWater_Lag + NAOw_Lag |
| 2 | 3 | Breakup + NAOw + OpenWater_Lag |
| 3 | 3 | Breakup + NAOw + NAOw_Lag |
| 4 | 3 | Breakup + OpenWater_Lag + NAOw_Lag |
| 5 | 3 | NAOw + OpenWater_Lag + NAOw_Lag |
| 6 | 2 | Breakup + NAOw |
| 7 | 2 | Breakup + OpenWater_Lag |
| 8 | 2 | Breakup + NAOw_Lag |
| 9 | 2 | NAOw + OpenWater_Lag |
| 10 | 2 | NAOw + NAOw_Lag |
| 11 | 2 | OpenWater_Lag + NAOw_Lag |
| 12 | 1 | Breakup |
| 13 | 1 | NAOw |
| 14 | 1 | OpenWater_Lag |
| 15 | 1 | NAOw_Lag |
| 16 | 4 | Breakup + AOw + OpenWater_Lag + AOw_Lag |
| 17 | 3 | Breakup + AOw + OpenWater_Lag |
| 18 | 3 | Breakup + AOw + AOw_Lag |
| 19 | 3 | Breakup + OpenWater_Lag + AOw_Lag |
| 20 | 3 | AOw + OpenWater_Lag + AOw_Lag |
| 21 | 2 | Breakup + AOw |
| 22 | 2 | Breakup + AOw_Lag |
| 23 | 2 | AOw + OpenWater_Lag |
| 24 | 2 | AOw + AOw_Lag |
| 25 | 2 | OpenWater_Lag + AOw_Lag |
| 26 | 1 | AOw |
| 27 | 1 | AOw_Lag |

Table S5. Pearson correlation matrix to test for collinearity of environmental variables. Variables that were correlated (*r* > |0.6|) are in bold and indicated with an asterisk (*) and were not included in the same multiple linear regression model. See Table S4 for descriptions of covariates. Correlated variables included: OpenWater and Breakup/Freeze-up, OpenWater_Lag and Breakup_Lag /Freeze-up_Lag, NAOw and AOw, and NAOw_Lag and AOw_Lag.

|  | Freeze-up | OpenWater | AOw | NAOw | Breakup_Lag | Freeze-up_Lag | OpenWater_Lag | AOw_Lag | NAOw_Lag |
| --- | --- | --- | --- | --- | --- | --- | --- | --- | --- |
| Breakup | -0.26 | **-0.88*** | -0.24 | -0.12 | 0.15 | -0.32 | -0.27 | -0.15 | -0.05 |
| Freeze-up |  | **0.69*** | -0.27 | -0.27 | -0.36 | 0.31 | 0.41 | 0.009 | -0.17 |
| OpenWater |  |  | 0.05 | -0.05 | -0.29 | 0.39 | 0.41 | 0.12 | -0.05 |
| AOw |  |  |  | **0.78*** | -0.02 | -0.005 | 0.01 | 0.10 | 0.06 |
| NAOw |  |  |  |  | -0.09 | -0.07 | 0.02 | 0.14 | 0.14 |
| Breakup_Lag |  |  |  |  |  | -0.33 | **-0.88*** | -0.29 | -0.13 |
| Freeze-up_Lag |  |  |  |  |  |  | **0.74*** | -0.24 | -0.28 |
| OpenWater_Lag |  |  |  |  |  |  |  | 0.08 | -0.05 |
| AOw_Lag |  |  |  |  |  |  |  |  | **0.77*** |

Table S6. Equations of the six-step process used to estimate total population energy density and storage energy in each year.

| Step | Description | Equation/Method |
| --- | --- | --- |
| 1 | Percentage of bears in each class | Percent adult males = Mean of bootstrapped percent adult males (number of adult males / total number of bears * 100) in five-year moving window |
| 2 | Annual number of bears | Annual number of bears = Draw a random value from a normal distribution of MARK estimates |
| 3 | Numbers of bears of each class | Number of adult males = Percent adult males * Annual number of bears |
| 4 | Mean energy for each class | Mean energy adult males = Mean of bootstrapped energy values for adult males |
| 5 | Total energy for each class | Total energy of adult males = Number of adult males * Mean energy adult males |
| 6 | Total yearly population energy | Total population energy = Total energy of adult males + Solitary adult females + Adult females with offspring + Subadult males + Subadult females + Yearlings + Cubs |

Table S7. Sample sizes of captures by Environment and Climate Change Canada (n), mean ± SE energy density, mean ± SE storage energy, and annual number of bears onshore in the core summering area ± SE (MARK estimate) for polar bears captured in western Hudson Bay, Canada from 1985 to 2018.

| Year | n | Energy density (MJ kg^-1^) | Storage energy (MJ) | MARK estimate |
| --- | --- | --- | --- | --- |
| 1985 | 53 | 21.5 ± 0.7 | 2640 ± 205 | 772 ± 213 |
| 1986 | 117 | 20.5 ± 0.6 | 2572 ± 125 | 1002 ± 212 |
| 1987 | 309 | 21.9 ± 0.4 | 2464 ± 85 | 1216 ± 77 |
| 1988 | 306 | 23.3 ± 0.4 | 2679 ± 83 | 1235 ± 64 |
| 1989 | 299 | 21.0 ± 0.4 | 2489 ± 82 | 1072 ± 56 |
| 1990 | 178 | 19.9 ± 0.5 | 2309 ± 105 | 1033 ± 60 |
| 1991 | 158 | 22.0 ± 0.5 | 2634 ± 121 | 1002 ± 58 |
| 1992 | 158 | 24.5 ± 0.6 | 2828 ± 127 | 948 ± 62 |
| 1993 | 139 | 24.9 ± 0.7 | 2554 ± 129 | 984 ± 61 |
| 1994 | 114 | 18.3 ± 0.5 | 2021 ± 119 | 1010 ± 63 |
| 1995 | 111 | 20.9 ± 0.6 | 2242 ± 127 | 1009 ± 84 |
| 1996 | 150 | 20.4 ± 0.5 | 2275 ± 112 | 964 ± 68 |
| 1997 | 187 | 19.8 ± 0.3 | 2136 ± 93 | 980 ± 63 |
| 1998 | 177 | 21.7 ± 0.4 | 2472 ± 99 | 859 ± 51 |
| 1999 | 130 | 23.0 ± 0.6 | 2570 ± 133 | 949 ± 57 |
| 2000 | 90 | 25.8 ± 0.7 | 3051 ± 172 | 829 ± 51 |
| 2001 | 117 | 22.5 ± 0.6 | 2375 ± 139 | 937 ± 73 |
| 2002 | 87 | 23.5 ± 0.6 | 2588 ± 174 | 897 ± 78 |
| 2003 | 153 | 21.8 ± 0.5 | 2452 ± 117 | 941 ± 74 |
| 2004 | 116 | 23.6 ± 0.6 | 2826 ± 144 | 961 ± 80 |
| 2005 | 91 | 25.0 ± 0.7 | 2481 ± 161 | 957 ± 96 |
| 2006 | 105 | 20.9 ± 0.5 | 2467 ± 143 | 913 ± 87 |
| 2007 | 80 | 22.3 ± 0.5 | 2628 ± 158 | 866 ± 78 |
| 2008 | 86 | 23.9 ± 0.6 | 2748 ± 151 | 848 ± 79 |
| 2009 | 111 | 21.3 ± 0.5 | 2414 ± 140 | 912 ± 76 |
| 2010 | 102 | 21.3 ± 0.6 | 2460 ± 145 | 826 ± 73 |
| 2011 | 99 | 19.9 ± 0.7 | 2280 ± 133 | 795 ± 72 |
| 2012 | 64 | 19.5 ± 0.6 | 2337 ± 173 | 843 ± 78 |
| 2013 | 68 | 19.9 ± 0.9 | 2437 ± 166 | 681 ± 64 |
| 2014 | 81 | 21.6 ± 0.8 | 2412 ± 157 | 649 ± 67 |
| 2015 | 68 | 19.8 ± 0.7 | 2124 ± 168 | 665 ± 67 |
| 2016 | 74 | 19.6 ± 0.7 | 2215 ± 141 | 642 ± 67 |
| 2017 | 89 | 21.3 ± 0.6 | 2532 ± 156 | 758 ± 69 |
| 2018 | 79 | 21.4 ± 0.7 | 2595 ± 144 | 617 ± 66 |

Table S8. Sample sizes (n) and the median energy density and storage energy (range) for each age/sex class of Western Hudson Bay polar bears from 1985 to 2018.

| Class | n | Energy density (MJ kg^-1^) | Storage energy (MJ) |
| --- | --- | --- | --- |
| Adult male | 1159 | 20.8 (5.6, 44.6) | 3895 (1156, 8206) |
| Solitary adult female | 540 | 30.3 (9.9, 57.1) | 3525 (1016, 6590) |
| Adult female with offspring | 807 | 19.8 (7.9, 44.1) | 2241 (916, 4736) |
| Subadult male | 296 | 21.3 (10.7, 57.0) | 2533 (948, 5541) |
| Subadult female | 331 | 20.6 (7.9, 45.6) | 1885 (794, 3857) |
| Yearling | 393 | 22.8 (6.8, 50.0) | 1457 (457, 3116) |
| Cub | 820 | 17.6 (4.7, 41.2) | 490 (79, 1694) |
| Total | 4346 | 20.8 (4.7, 57.1) | 2370 (79, 8206) |

Table S9. Summary of generalized additive mixed models for energy density over time for each age/sex class. Note: the linear mixed effects model was either the best model or was within 4 AIC of the generalized additive mixed model for solitary adult female, subadult female, and yearling (Table S3) and the linear model summaries are therefore included as well. Bold and * indicates significant (*p* ≤ 0.05). edf: effective degrees of freedom (where an edf of 1.0 indicates a linear trend and an edf > 1.0 indicates a non-linear trend).

| Class | | Parametric coefficients: Intercept | | | |
| --- | --- | --- | --- | --- | --- |
|  |  | Estimate | SE | t-value | p-value |
| Adult male | | 20.952 | 0.170 | 123.400 | **<0.001*** |
| Solitary adult female | | 30.716 | 0.373 | 82.460 | **<0.001*** |
| Adult female with offspring | | 20.329 | 0.192 | 105.700 | **<0.001*** |
| Subadult male | | 21.741 | 0.323 | 67.390 | **<0.001*** |
| Subadult female | | 21.010 | 0.291 | 72.180 | **<0.001*** |
| Yearling | | 22.989 | 0.294 | 78.180 | **<0.001*** |
| Cub | | 18.230 | 0.171 | 106.600 | **<0.001*** |
| Class | | Approximate significance of smooth terms: s(Year) | | | |
|  |  | edf |  | F-value | p-value |
| Adult male | | 7.025 |  | 7.289 | **<0.001*** |
| Solitary adult female | | 1.000 |  | 22.880 | **<0.001*** |
| Adult female with offspring | | 5.951 |  | 8.588 | **<0.001*** |
| Subadult male | | 5.369 |  | 5.602 | **<0.001*** |
| Subadult female | | 1.364 |  | 0.060 | 0.873 |
| Yearling | | 1.860 |  | 1.285 | 0.358 |
| Cub | | 7.878 |  | 7.648 | **<0.001*** |
| Linear models | | Estimate | SE | t-value | p-value |
| Solitary adult female | Intercept | 377.538 | 72.521 | 5.206 | **<0.001*** |
|  | Year | -0.174 | 0.036 | -4.783 | **<0.001*** |
| Subadult female | Intercept | 20.240 | 63.814 | 0.317 | 0.751 |
|  | Year | 0.0004 | 0.032 | 0.012 | 0.990 |
| Yearling | Intercept | 73.116 | 62.969 | 1.161 | 0.246 |
|  | Year | -0.025 | 0.032 | -0.796 | 0.426 |

Table S10. Summary of generalized additive mixed models for storage energy over time for each age/sex class. Note: the linear mixed effects model was either the best model or was within 4 AIC of the generalized additive model for solitary adult female and subadult female (Table S3) and the linear model summaries are therefore included as well. Bold and * indicates significant (*p* ≤ 0.05). edf: effective degrees of freedom (where an edf of 1.0 indicates a linear trend and an edf > 1.0 indicates a non-linear trend).

| Class | | Parametric coefficients: Intercept | | | |
| --- | --- | --- | --- | --- | --- |
|  |  | Estimate | SE | t-value | p-value |
| Adult male | | 3903.660 | 35.550 | 109.800 | **<0.001*** |
| Solitary adult female | | 3558.350 | 43.670 | 81.470 | **<0.001*** |
| Adult female with offspring | | 2335.110 | 22.040 | 106.000 | **<0.001*** |
| Subadult male | | 2577.580 | 44.090 | 58.470 | **<0.001*** |
| Subadult female | | 1944.88 | 30.510 | 63.740 | **<0.001*** |
| Yearling | | 1498.700 | 21.900 | 68.430 | **<0.001*** |
| Cub | | 521.595 | 6.849 | 76.160 | **<0.001*** |
| Class | | Approximate significance of smooth terms: s(Year) | | | |
|  |  | edf |  | F-value | p-value |
| Adult male | | 7.349 |  | 4.105 | **<0.001*** |
| Solitary adult female | | 1.000 |  | 29.780 | **<0.001*** |
| Adult female with offspring | | 6.919 |  | 6.839 | **<0.001*** |
| Subadult male | | 3.404 |  | 6.236 | **<0.001*** |
| Subadult female | | 1.369 |  | 0.868 | 0.486 |
| Yearling | | 6.292 |  | 2.984 | **0.007*** |
| Cub | | 8.227 |  | 7.570 | **<0.001*** |
| Linear models | | Estimate | SE | t-value | p-value |
| Solitary adult female | Intercept | 49988.720 | 8509.483 | 5.874 | **<0.001*** |
|  | Year | -23.240 | 4.259 | -5.457 | **<0.001*** |
| Subadult female | Intercept | 7633.328 | 6692.350 | 1.141 | 0.255 |
|  | Year | -2.848 | 3.350 | -0.850 | 0.401 |

Table S11. Summary of generalized additive models for the percentage of the total population storage energy contributed by each age/sex class over time. Note: the linear model was either the best model or was within 4 AIC of the generalized additive model for adult male, subadult male, subadult female, yearling, and cub (Table S3) and the linear model summaries are therefore included as well. Bold and * indicates significant (*p* ≤ 0.05). edf: effective degrees of freedom (where an edf of 1.0 indicates a linear trend and an edf > 1.0 indicates a non-linear trend).

| Class | | Parametric coefficients: Intercept | | | |
| --- | --- | --- | --- | --- | --- |
|  |  | Estimate | SE | t-value | p-value |
| Adult male | | 43.763 | 1.181 | 37.060 | **<0.001*** |
| Solitary adult female | | 16.732 | 0.893 | 18.730 | **<0.001*** |
| Adult female with offspring | | 17.640 | 0.569 | 31.000 | **<0.001*** |
| Subadult male | | 6.659 | 0.486 | 13.700 | **<0.001*** |
| Subadult female | | 5.880 | 0.509 | 11.550 | **<0.001*** |
| Yearling | | 5.355 | 0.437 | 12.260 | **<0.001*** |
| Cub | | 3.972 | 0.184 | 21.540 | **<0.001*** |
| Class | | Approximate significance of smooth terms: s(Year) | | | |
|  |  | edf |  | F-value | p-value |
| Adult male | | 3.084 |  | 3.917 | **0.016*** |
| Solitary adult female | | 4.346 |  | 5.422 | **0.002*** |
| Adult female with offspring | | 2.674 |  | 3.293 | **0.029*** |
| Subadult male | | 1.903 |  | 3.886 | **0.021*** |
| Subadult female | | 1.000 |  | 3.499 | 0.070 |
| Yearling | | 1.265 |  | 0.095 | 0.863 |
| Cub | | 2.413 |  | 2.844 | **0.049*** |
| Linear models | | Estimate | SE | t-value | p-value |
| Adult male | Intercept | -587.604 | 262.100 | -2.242 | **0.032*** |
|  | Year | 0.315 | 0.131 | 2.409 | **0.021*** |
| Subadult male | Intercept | 270.500 | 102.628 | 2.636 | **0.013*** |
|  | Year | -0.132 | 0.051 | -2.571 | **0.015*** |
| Subadult female | Intercept | 200.219 | 103.894 | 1.927 | 0.063 |
|  | Year | -0.097 | 0.052 | -1.871 | 0.071 |
| Yearling | Intercept | -6.562 | 89.536 | -0.073 | 0.942 |
|  | Year | 0.006 | 0.045 | 0.133 | 0.895 |
| Cub | Intercept | 42.970 | 41.456 | 1.037 | 0.308 |
|  | Year | -0.019 | 0.021 | -0.941 | 0.354 |

Table S12. The best linear mixed effects models for energy density and the environmental covariates for each polar bear age/sex class in Western Hudson Bay from 1985 to 2018. The model F-statistic, R^2^, β coefficients (β), standard error (SE), and p-values (*p*) are included. Bold and * indicates significant (*p* ≤ 0.05). Model number corresponds to Table S4.

| Class | Model no. | Covariates | Intercept β | β | SE | *p* |
| --- | --- | --- | --- | --- | --- | --- |
| Adult male | 22 | **Breakup** | 12.962 | 0.047 | 0.013 | **<0.001*** |
|  |  | **AOw_Lag** |  | -0.267 | 0.122 | **0.029*** |
| Solitary adult female | 6 | **Breakup** | 7.538 | 0.133 | 0.027 | **<0.001*** |
|  |  | **NAOw** |  | 0.473 | 0.145 | **0.001*** |
| Adult female with offspring | 27 | **AOw_Lag** | 20.333 | -0.407 | 0.156 | **0.010*** |
| Subadult male | 7 | **Breakup** | -6.983 | 0.115 | 0.029 | **<0.001*** |
|  |  | **OpenWater_Lag** |  | 0.073 | 0.021 | **0.001*** |
| Subadult female | 24 | **AOw** | 21.082 | -0.553 | 0.238 | **0.026*** |
|  |  | **AOw_Lag** |  | 0.594 | 0.249 | **0.023*** |
| Yearling | 12 | **Breakup** | 11.682 | 0.066 | 0.026 | **0.012*** |
| Cub | 4 | **Breakup** | 0.544 | **0.092** | **0.016** | **<0.001*** |
|  |  | OpenWater_Lag |  | 0.018 | 0.012 | 0.152 |
|  |  | NAOw_Lag |  | -0.148 | 0.079 | 0.059 |

Table S13. Linear mixed effects model results for energy density with sea ice breakup and previous open water period for each polar bear age/sex class in Western Hudson Bay from 1985 to 2018. The model F-statistic, R^2^, β coefficients (β), standard error (SE), and *p*-values (*p*) are included. Bold and * indicates significant (*p* ≤ 0.05). Model number corresponds to Table S4.

| Class | Model no. | Covariates | Intercept β | Β | SE | *p* |
| --- | --- | --- | --- | --- | --- | --- |
| Adult male | 7 | **Breakup** | 7.430 | 0.062 | 0.012 | **<0.001*** |
|  |  | **OpenWater_Lag** |  | 0.023 | 0.010 | **0.015*** |
| Solitary adult female | 7 | **Breakup** | 24.862 | 0.083 | 0.030 | **0.007*** |
|  |  | **OpenWater_Lag** |  | -0.067 | 0.026 | **0.010*** |
| Adult female with offspring | 7 | **Breakup** | 9.243 | 0.045 | 0.017 | **0.007*** |
|  |  | **OpenWater_Lag** |  | 0.027 | 0.013 | **0.034*** |
| Subadult male | 7 | **Breakup** | -6.983 | 0.115 | 0.029 | **<0.001*** |
|  |  | **OpenWater_Lag** |  | 0.073 | 0.021 | **0.001*** |
| Subadult female | 7 | **Breakup** | 9.047 | 0.053 | 0.025 | **0.041*** |
|  |  | OpenWater_Lag |  | 0.024 | 0.021 | 0.262 |
| Yearling | 7 | **Breakup** | 10.310 | 0.070 | 0.029 | **0.017*** |
|  |  | OpenWater_Lag |  | 0.006 | 0.021 | 0.768 |
| Cub | 7 | **Breakup** | -0.446 | 0.096 | 0.016 | **<0.001*** |
|  |  | OpenWater_Lag |  | 0.019 | 0.012 | 0.121 |

Table S14. The best linear mixed effects models for storage energy and the environmental covariates for each polar bear age/sex class in Western Hudson Bay from 1985 to 2018. The model F-statistic, R^2^, β coefficients (β), standard error (SE), and p-values (*p*) are included. Bold and * indicates significant (*p* ≤ 0.05). Model number corresponds to Table S4.

| Class | Model no. | Covariates | Intercept β | β | SE | *p* |
| --- | --- | --- | --- | --- | --- | --- |
| Adult male | 16 | **Breakup** | 1156.352 | 13.579 | 2.724 | **<0.001*** |
|  |  | AOw |  | 20.501 | 24.335 | 0.400 |
|  |  | OpenWater_Lag |  | 3.435 | 1.961 | 0.080 |
|  |  | AOw_Lag |  | -12.896 | 24.758 | 0.603 |
| Solitary adult female | 1 | **Breakup** | 2387.462 | 12.209 | 3.612 | **0.001*** |
|  |  | **NAOw** |  | 50.883 | 17.373 | **0.004*** |
|  |  | **OpenWater_Lag** |  | -8.251 | -2.737 | **0.007*** |
|  |  | **NAOw_Lag** |  | 43.037 | 17.910 | **0.018*** |
| Adult female with offspring | 16 | **Breakup** | 1090.710 | 5.794 | 2.042 | **0.005*** |
|  |  | AOw |  | -8.834 | 18.157 | 0.627 |
|  |  | OpenWater_Lag |  | 2.053 | 1.458 | 0.160 |
|  |  | AOw_Lag |  | -27.719 | 18.564 | 0.137 |
| Subadult male | 16 | **Breakup** | -1976.698 | 20.525 | 4.636 | **<0.001*** |
|  |  | AOw |  | 47.046 | 37.444 | 0.217 |
|  |  | **OpenWater_Lag** |  | 8.498 | 3.168 | **0.011*** |
|  |  | AOw_Lag |  | 28.103 | 41.642 | 0.504 |
| Subadult female | 16 | **Breakup** | 774.275 | 7.290 | 3.037 | **0.022*** |
|  |  | AOw |  | -32.318 | 27.962 | 0.256 |
|  |  | OpenWater_Lag |  | -0.488 | 2.250 | 0.830 |
|  |  | **AOw_Lag** |  | 79.896 | 27.887 | **0.007*** |
| Yearling | 12 | **Breakup** | 477.791 | 5.958 | 2.000 | **0.003*** |
| Cub | 17 | **Breakup** | -519.882 | 5.515 | 0.670 | **<0.001*** |
|  |  | **AOw** |  | 17.480 | 6.132 | **0.004*** |
|  |  | OpenWater_Lag |  | 0.807 | 0.489 | 0.099 |

Table S15. Linear mixed effects model results for storage energy with sea ice breakup and previous open water period for each polar bear age/sex class in Western Hudson Bay from 1985 to 2018. The model F-statistic, R^2^, β coefficients (β), standard error (SE), and *p*-values (*p*) are included. Bold and * indicates significant (*p* ≤ 0.05). Model number corresponds to Table S4.

| Class | Model no. | Covariates | Intercept β | β | SE | *p* |
| --- | --- | --- | --- | --- | --- | --- |
| Adult male | 7 | **Breakup** | 1280.868 | 532.002 | 2.408 | **<0.001*** |
|  |  | OpenWater_Lag |  | 3.089 | 1.922 | 0.109 |
| Solitary adult female | 7 | **Breakup** | 3263.070 | 8.726 | 3.560 | **0.016*** |
|  |  | **OpenWater_Lag** |  | -9.675 | 3.013 | **0.002*** |
| Adult female with offspring | 7 | **Breakup** | 912.626 | 6.837 | 1.889 | **<0.001*** |
|  |  | OpenWater_Lag |  | 2.044 | 1.440 | 0.157 |
| Subadult male | 7 | **Breakup** | -1315.877 | 17.417 | 4.102 | **<0.001*** |
|  |  | **OpenWater_Lag** |  | 7.505 | 2.995 | **0.016*** |
| Subadult female | 7 | **Breakup** | 645.888 | 6.755 | 2.659 | **0.015*** |
|  |  | OpenWater_Lag |  | 1.248 | 2.181 | 0.571 |
| Yearling | 7 | **Breakup** | 467.336 | 5.985 | 2.200 | **0.007*** |
|  |  | OpenWater_Lag |  | 0.047 | 1.602 | 0.976 |
| Cub | 7 | **Breakup** | -375.534 | 4.834 | 0.629 | **<0.001*** |
|  |  | OpenWater_Lag |  | 0.597 | 0.486 | 0.219 |

Table S16. Dunn’s test comparing energy density among age/sex classes of western Hudson Bay polar bears from 1985 to 2018. Bold and * indicates significant (*p* ≤ 0.05).

|  | Adult male (1159) | Solitary adult female  (540) | Adult female with offspring (807) | Subadult male  (296) | Subadult female  (331) | Yearling  (393) | Cub (820) |
| --- | --- | --- | --- | --- | --- | --- | --- |
| Adult male |  | **<0.001*** | **<0.001*** | 0.073 | 0.431 | **<0.001*** | **<0.001*** |
| Solitary adult female |  |  | **<0.001*** | **<0.001*** | **<0.001*** | **<0.001*** | **<0.001*** |
| Adult female with offspring |  |  |  | **<0.001*** | **0.012*** | **<0.001*** | **<0.001*** |
| Subadult male |  |  |  |  | 0.094 | **0.002*** | **<0.001*** |
| Subadult female |  |  |  |  |  | **<0.001*** | **<0.001*** |
| Yearling |  |  |  |  |  |  | **<0.001*** |

Table S17. Dunn’s test comparing storage energy among age/sex classes of western Hudson Bay polar bears from 1985 to 2018. Bold and * indicates significant (*p* ≤ 0.05).

|  | Adult male (1159) | Solitary adult female  (540) | Adult female with offspring (807) | Subadult male  (296) | Subadult female  (331) | Yearling  (393) | Cub (820) |
| --- | --- | --- | --- | --- | --- | --- | --- |
| Adult male |  | **<0.001*** | **<0.001*** | **<0.001*** | **<0.001*** | **<0.001*** | **<0.001*** |
| Solitary adult female |  |  | **<0.001*** | **<0.001*** | **<0.001*** | **<0.001*** | **<0.001*** |
| Adult female with offspring |  |  |  | **0.002*** | **<0.001*** | **<0.001*** | **<0.001*** |
| Subadult male |  |  |  |  | **<0.001*** | **<0.001*** | **<0.001*** |
| Subadult female |  |  |  |  |  | **<0.001**** | **<0.001*** |
| Yearling |  |  |  |  |  |  | **<0.001*** |

Table S18. Summary of generalized additive models for population energy density and storage energy over time. Bold and * indicates significant (*p* ≤ 0.05). edf: effective degrees of freedom (where an edf of 1.0 indicates a linear trend and an edf > 1.0 indicates a non-linear trend).

| Energy metric |  | | | |
| --- | --- | --- | --- | --- |
| Energy density | Parametric coefficients: Intercept | | | |
|  | Estimate | SE | t-value | p-value |
|  | 19944.800 | 265.600 | 75.100 | **<0.001*** |
|  | Approximate significance of smooth terms: s(Year) | | | |
|  | edf |  | F-value | p-value |
|  | 4.865 |  | 44.250 | **<0.001*** |
| Storage energy | Parametric coefficients: Intercept | | | |
|  | Estimate | SE | t-value | p-value |
|  | 2270218 | 29511 | 76.930 | **<0.001*** |
|  | Approximate significance of smooth terms: s(Year) | | | |
|  | edf |  | F-value | p-value |
|  | 5.173 |  | 49.340 | **<0.001*** |

Table S19. Model selection for total population energy density and storage energy for Western Hudson Bay polar bears from 1985 to 2018. The top four models for each energy variable are shown with the associated Akaike Information Criterion (AIC), ΔAIC (AIC difference between each model and the best model), and Akaike weight (*w_i_*). The best model is indicated in bold. Model number corresponds to Table S4.

| Response | Model no. | Covariates | AIC | ΔAIC | *w_i_* |
| --- | --- | --- | --- | --- | --- |
| **Energy density** | **7** | **Breakup + OpenWater_Lag** | **645.007** | **0** | **0.43** |
| Energy density | 2 | Breakup + NAOw + OpenWater_Lag | 646.453 | 1.446 | 0.21 |
| Energy density | 17 | Breakup + AOw + OpenWater_Lag | 646.569 | 1.562 | 0.20 |
| Energy density | 19 | Breakup + OpenWater_Lag + AOw_Lag | 646.965 | 1.958 | 0.16 |
| **Storage energy** | **7** | **Breakup + OpenWater_Lag** | **967.551** | **0** | **0.45** |
| Storage energy | 2 | Breakup + NAOw + OpenWater_Lag | 969.175 | 1.624 | 0.20 |
| Storage energy | 17 | Breakup + AOw + OpenWater_Lag | 969.276 | 1.725 | 0.19 |
| Storage energy | 19 | Breakup + OpenWater_Lag + AOw_Lag | 969.488 | 1.937 | 0.17 |

**Supplementary Figures**


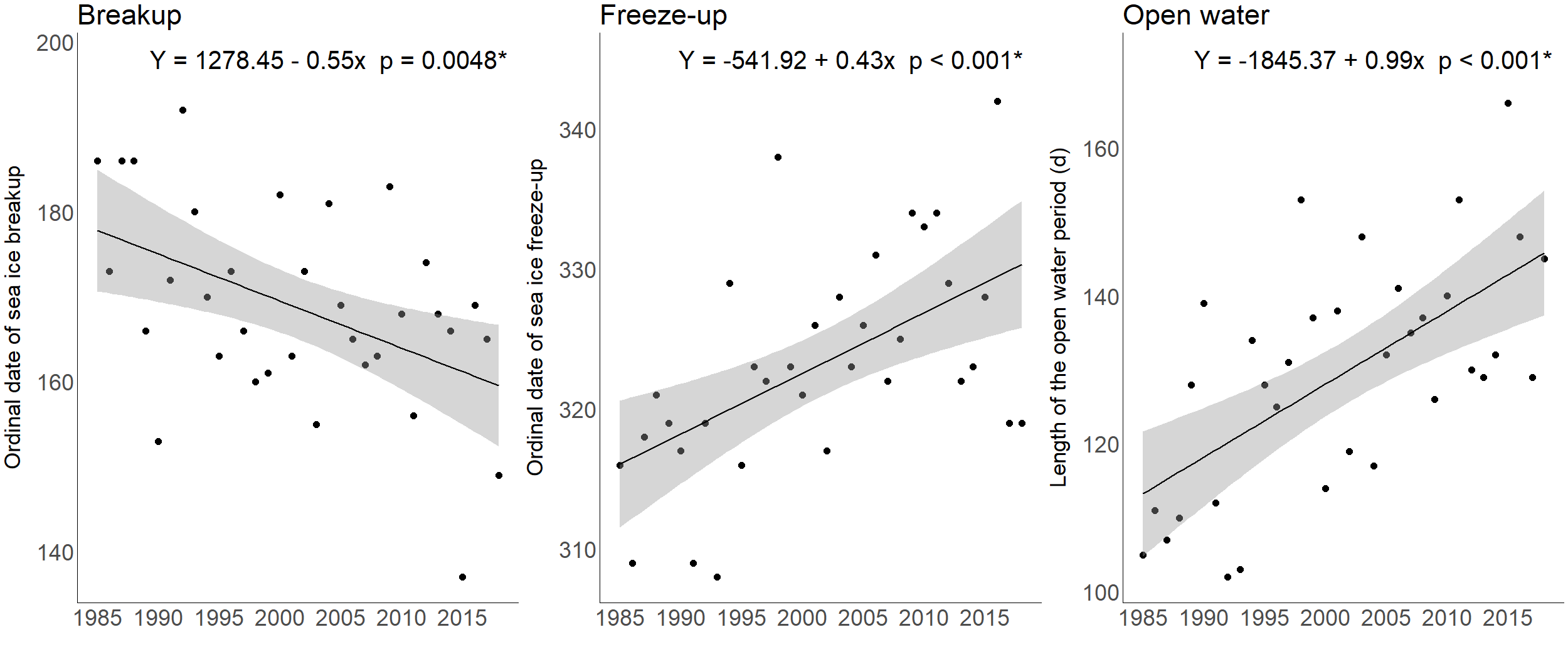
Figure S1. Linear regressions (black line) with 95% confidence intervals (grey) for the temporal dynamics of sea ice breakup, freeze-up, and the length of the open water period in western Hudson Bay from 1985 to 2018.


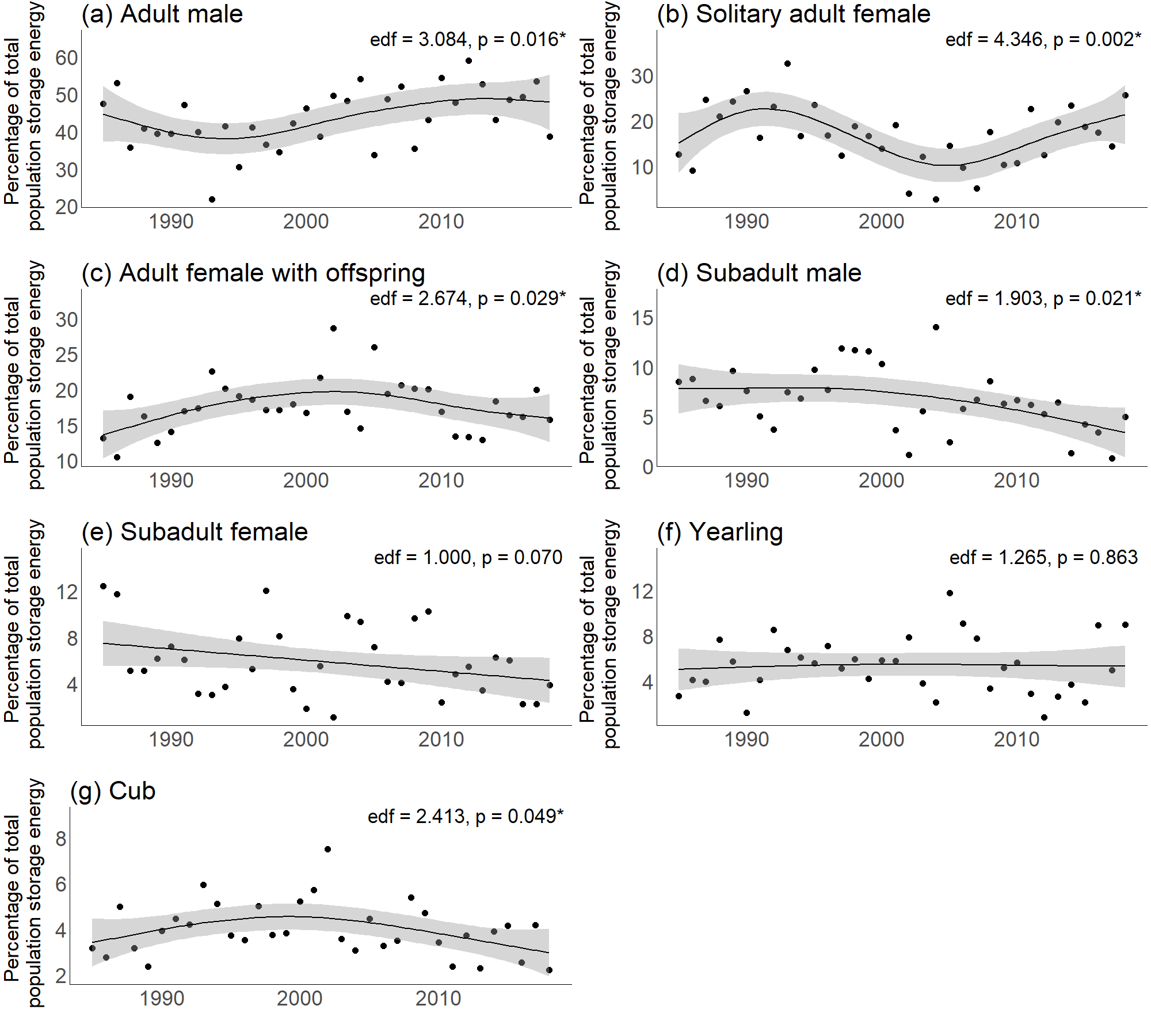
Figure S2. Generalized additive models (black line) with 95% confidence intervals (grey) for the percentage of the total population storage energy contributed by each age/sex class over time for Western Hudson Bay polar bears. See Table S11 for model summaries.


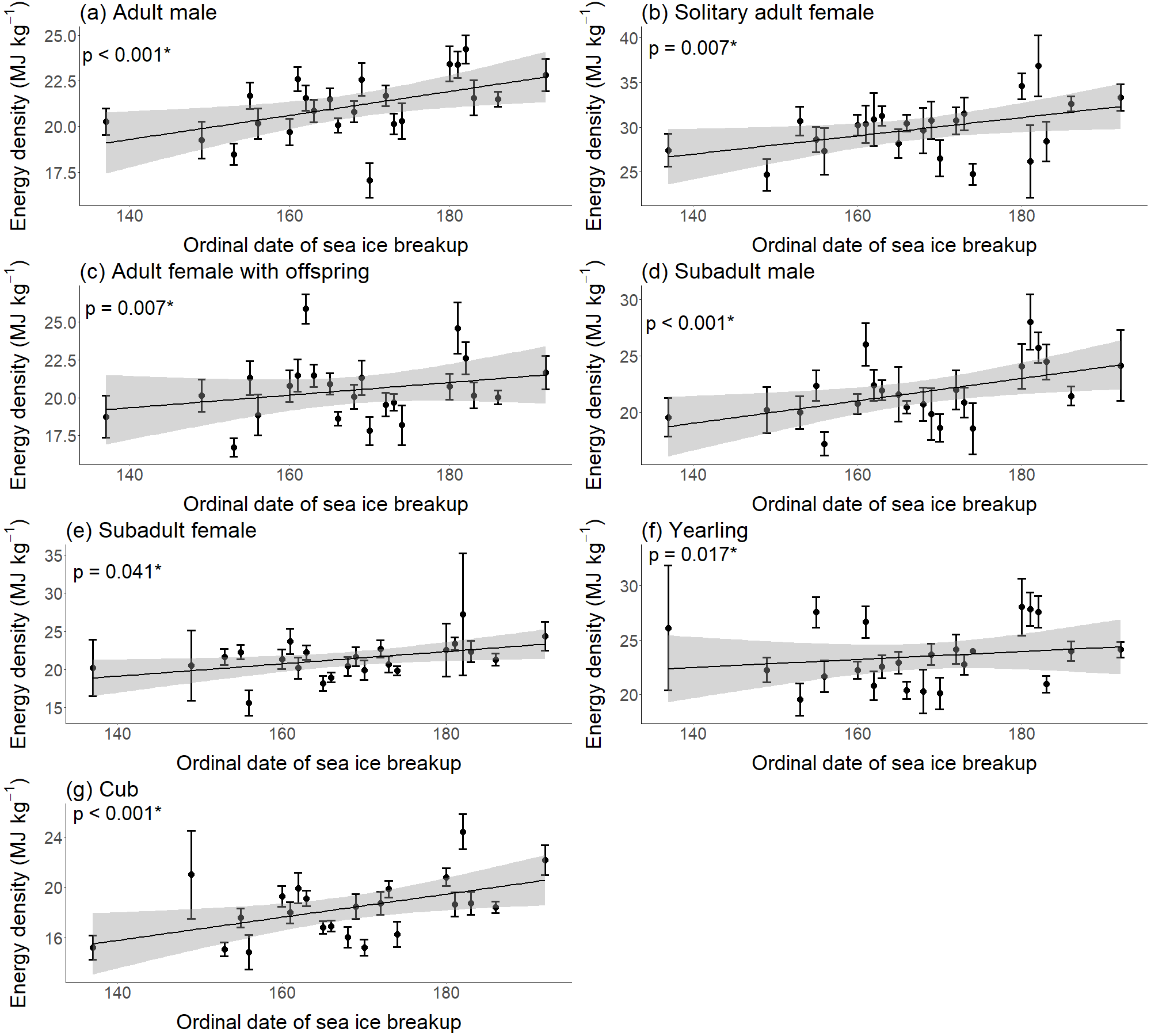
Figure S3. Linear regressions (black line) with 95% confidence intervals (grey) for energy density (mean ± standard error) with the date of sea ice breakup for each age/sex class of Western Hudson Bay polar bears. See Table S13 for model summaries.


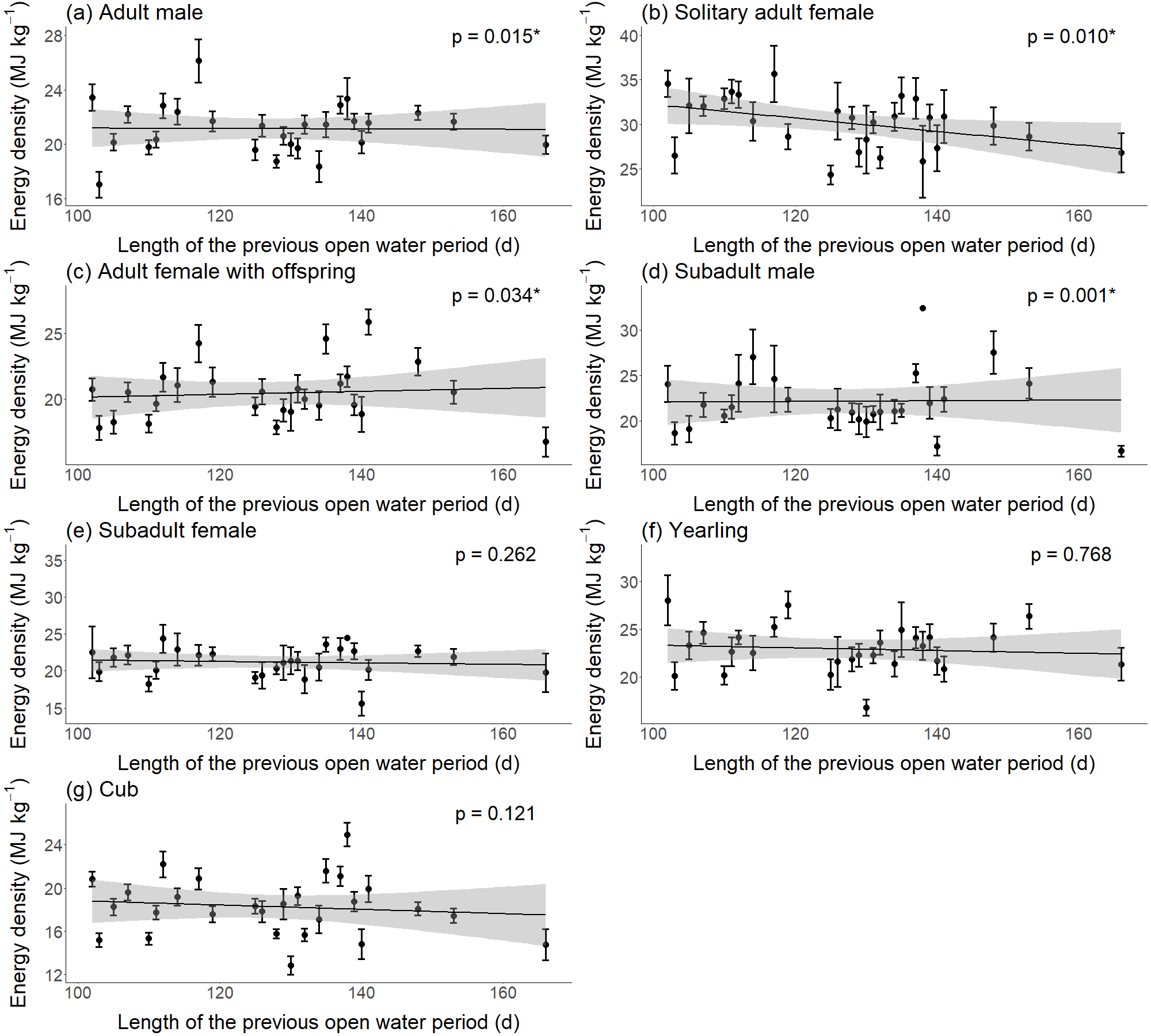
Figure S4. Linear regressions (black line) with 95% confidence intervals (grey) for energy density (mean ± standard error) with the length of the previous open water period for each age/sex class of Western Hudson Bay polar bears. See Table S13 for model summaries.


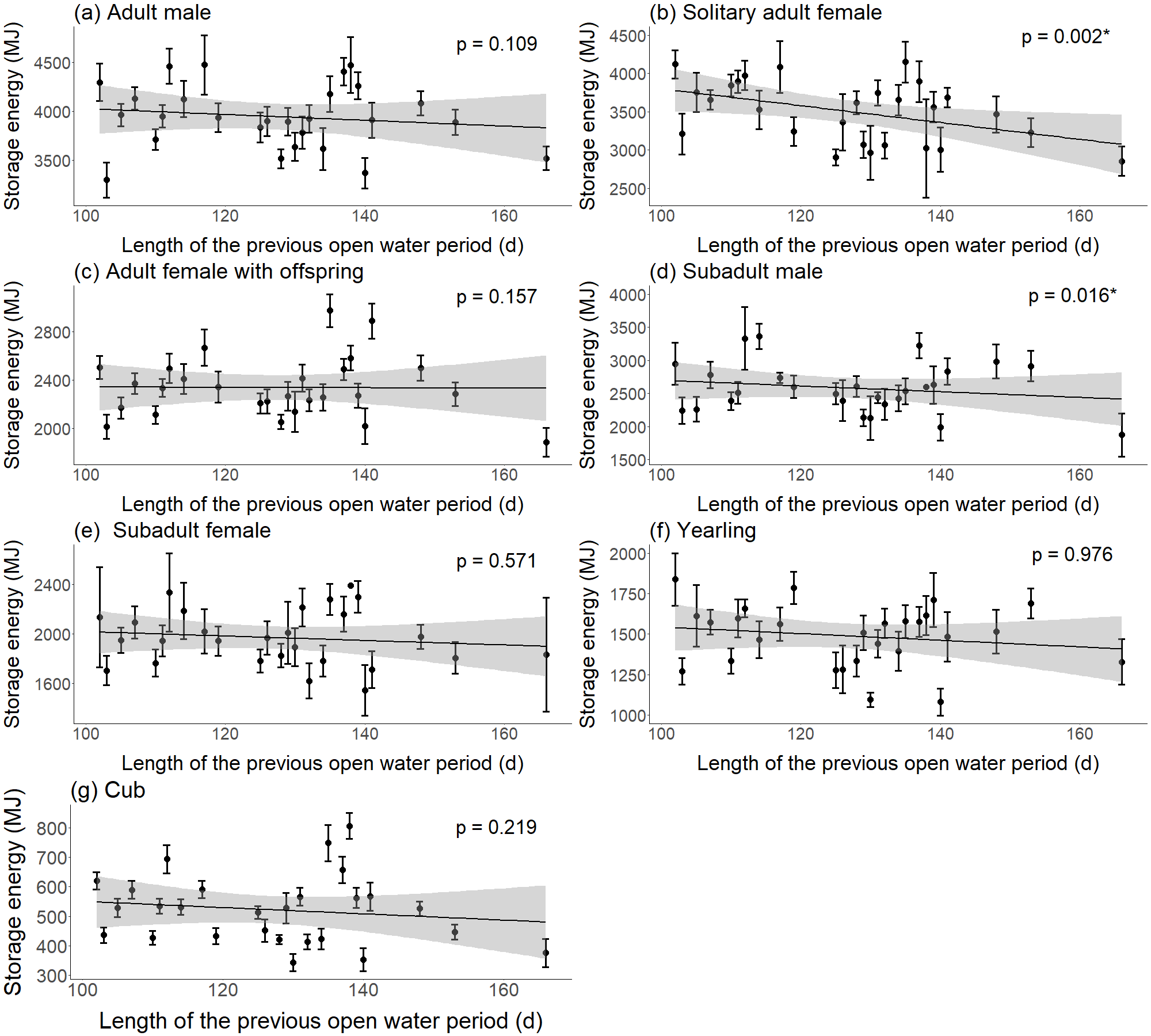
Figure S5. Linear regressions (black line) with 95% confidence intervals (grey) for storage energy (mean ± standard error) with the length of the previous open water period for each age/sex class of Western Hudson Bay polar bears. See Table S15 for model summaries.
